# Full Genome Sequence of a Western Reference Strain of Bluetongue Virus Serotype 16 from Nigeria

**DOI:** 10.1128/genomeA.00684-13

**Published:** 2013-09-19

**Authors:** Peter P. C. Mertens, Narender S. Maan, Manjunatha N. Belaganahalli, Karam Pal Singh, Kyriaki Nomikou, Sushila Maan

**Affiliations:** Vector-borne Diseases Programme, the Pirbright Institute, Woking, Surrey, United Kingdoma; College of Veterinary Sciences, LLR University of Veterinary and Animal Sciences, Hisar, Haryana, Indiab; Pathology Laboratory, Centre for Animal Disease Research and Diagnosis, Indian Veterinary Research Institute, Izatnagar, Indiac

## Abstract

The genome of NIG1982/10, a Nigerian bluetongue virus serotype 16 (BTV-16) strain, was sequenced (19,193 bp). Comparisons to BTV strains from other areas of the world show that all 10 genome segments of NIG1982/10 are derived from a western lineage (w), indicating that it represents a suitable reference strain of BTV-16w.

## GENOME ANNOUNCEMENT

Bluetongue virus (BTV) is a double-stranded RNA virus (genus *Orbivirus*, family *Reoviridae*) (1, 2) that infects domesticated and wild ruminants. The virus is transmitted between mammalian hosts by vector-competent *Culicoides* species, and it can be transmitted vertically in ruminants or by ingestion of infected tissues. Bluetongue (BT) represents a significant threat to livestock industries worldwide. The recent emergence of 10 BTV serotypes (since 1998) and massive outbreaks in Europe caused by BTV serotype 8, western lineage (BTV-8w) (2006 to 2009), illustrate the threat posed by emerging arboviral diseases (3).

The BTV capsid is icosahedral and nonenveloped, composed of three concentric protein layers (4). The 10 linear double-stranded RNA (dsRNA) genome segments (Seg-1 to Seg-10) encode 7 structural proteins (VP1 to VP7) and at least 4 nonstructural proteins (NS1, NS2, NS3/3a, and NS4) (5, 6). VP2 and VP5 (encoded by Seg-2 and Seg-6, respectively) are the most variable BTV proteins. They form the outer capsid, representing a target for neutralizing antibodies (particularly VP2), and determine virus serotype (26 known serotypes) (7, 8).

We report the complete genome sequencing of a 1982 Nigerian BTV-16 isolate (NIG1982/10) provided by the Onderstepoort Veterinary Research Institute, South Africa (http://www.reoviridae.org/dsRNA_virus_proteins/ReoID/virus-nos-by-country.htm). The NIG1982/10 genome was converted to cDNA by full-length amplification of cDNAs (FLAC) and sequenced as described elsewhere (9).

The NIG1982/10 genome comprises 19,193 bp, with Seg-1 to Seg-10 being 3,944, 2,935, 2,772, 1,981, 1,773, 1,637, 1,156, 1,125, 1,048, and 822 bp, respectively. The structural proteins are encoded by Seg-1 (VP1), 1,302 amino acids (aa); Seg-2 (VP2), 959 aa; Seg-3 (VP3), 901 aa; Seg-4 (VP4), 644 aa; Seg-6 (VP5), 526 aa; Seg-7 (VP7), 349 aa; and Seg-9 (VP6), 329 aa. The nonstructural proteins are encoded by Seg-5 (NS1), 552 aa; Seg-8 (NS2), 354 aa; Seg-9 (NS4), 77 aa; and Seg-10 (NS3/NS3a), 229/216 aa.

Four eastern BTV-16 strains (Indian, Chinese, Australian, and a South African reference strain) have been sequenced (10–13). All 10 genome segments of the reference, vaccine, and Chinese strains (RSArrrr/16, RSAvvvv/16, and BN96/16) (13) are derived from a very recent common ancestor, showing >99% sequence identity. A BTV-16 strain from Australia (strain DPP96) represents a distinct virus lineage within the major eastern topotype (e). The Indian strain of BTV-16 is a reassortant, showing high identity levels (92 to 97%) to RSArrrr/16 in most genome segments but containing a western topotype (w) Seg-5 (89% nucleotide [nt] identity).

All 10 genome segments of NIG1982/10 are derived from a western lineage, with only 77% to 84% nt identity to the eastern topotype reference strain RSArrrr/16 from Pakistan and 76.4% to 83% identity to the Australian BTV-16e strain (DPP96). Seg-2 (VP2) and Seg-6 (VP5) of NIG1982/10 show up to 95% nt (97% aa) and 97% nt (100% aa) identities, respectively, to other BTV-16w strains, and the conserved genome segments (Seg-1, 3, 4, 5, 7, 8, and 9) of NIG1982/10 show >85.4% identity to other western topotype BTVs. The topotype of Seg-10 from NIG1982/10 is less clear-cut, with ≥81.8% and 81.2% to 84.5% nt identities to BTVw and BTVe strains, respectively.

We conclude that NIG1982/10 is suitable as a reference strain of BTV-16w for serological, phylogenetic, and molecular epidemiology studies. The use of full-genome sequencing for the characterization of novel BTV isolates helps to identify virus topotype, as well as cross-topotype reassortant viruses generated in the field.

### Nucleotide sequence accession numbers.

The nucleotide sequences for NIG1982/10 are deposited in GenBank under accession no. AJ585150 (Seg-2), DQ186819 (Seg-3), AJ586694 (Seg-6), and KC853052 to KC853058 for Seg-1, Seg-4, Seg-5/NS1, and Seg-7 to Seg-10, respectively.
